# An Unusual Case of Nonhealing Granulomatous Keratitis Caused by *Mycobacterium chelonae* in a Healthy Middle Aged Adult

**DOI:** 10.1155/2015/708312

**Published:** 2015-12-22

**Authors:** Vipul Bhandari, Sriganesh, Kirti Relekar

**Affiliations:** Department of Cornea, Nethradhama Eye Hospital, Bengaluru 560070, India

## Abstract

*Purpose*. To report a rare presentation of culture positive* Mycobacterium chelonae* (*M. chelonae*) corneal ulcer and its management.* Case Report*. We report a rare case with history of chronic pain and blurriness of vision. Examination revealed chronic nonhealing paracentral corneal ulcer inferiorly at 5 to 7 o'clock meridian with anterior chamber cells 1+ unresponsive to routine antibiotic and antifungal medications with Mantoux test (MT) positivity in a middle aged nondiabetic patient with no prior obvious history of trauma, ocular surgery, and contact lens usage.* Discussion*. Ziehl Neelsen (ZN) staining in nonhealing ulcer revealed acid fast bacilli typical of* M. chelonae* with subsequent culture positivity in Lowenstein Jensen (LJ) medium. Subsequent treatment with topical fortified amikacin and tobramycin resulted in rapid healing of corneal ulcer.* Conclusion. M. chelonae* presenting as a chronic nonhealing corneal ulcer spontaneously occurring in a healthy young adult with no predisposing factor draws the need to have a good index of suspicion by performing ZN stain and culture and its subsequent successful management with topical fortified amikacin and tobramycin.

## 1. Introduction

The nontuberculous* Mycobacterium chelonae* (*M. chelonae*) is an omnipresent saprophyte present in soil, water, and air [1]. In the eye, the organism is known to cause dacryocystitis, canaliculitis, conjunctivitis, scleritis, endophthalmitis, and keratitis [2, 3]. Risk factors for infection include both accidental and surgical trauma, laser in situ keratomileusis (LASIK), penetrating keratoplasty (PK), and all procedures involving retained biomaterial [2, 3]. Breakdown of the corneal epithelium due to surgical trauma increases the risk of surface infection and increases the virulence of these rapidly growing mycobacteria [4]. Nonhealing corneal ulcers unresponsive to routine antibacterial, antifungal agents with normal corneal sensations are usually associated with underlying diabetes mellitus and systemic immunocompromised states. Nocardia and atypical mycobacteria like* M. chelonae* and* M. fortuitum* and scrofuloderma have been identified as uncommon infective causes of chronic nonhealing corneal ulcers [5]. A history of trauma with foreign body (usually metallic), prior ocular surgery, or contact lens usage is usually present in such cases [6]. Local iatrogenic insult or systemic immunosuppression has been identified as the most common cause of nontuberculous mycobacterial associated ocular infections [6]. Due to frequent delay in diagnosis,* M. chelonae* being rapid growers lead to significant blinding complications [6]. Spontaneous corneal infection with the* Bacillus* occurring in the absence of any predisposing factor in a healthy young adult has not been reported till date. Herein we report a very rare case of nonhealing corneal ulcer with Ziehl Neelsen (ZN) stain and culture positivity for* M. chelonae* and describe its management.

## 2. Case Report

**Figure 1:**
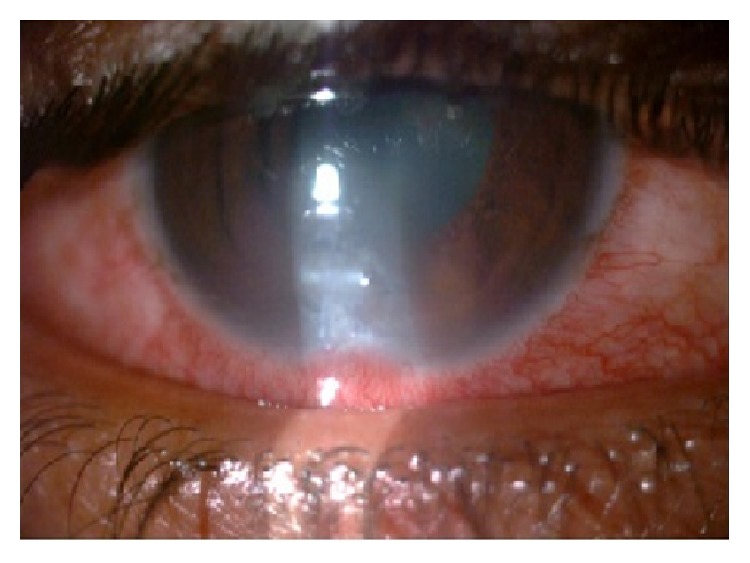
Corneal ulcer-paracentral, whitish dry looking ulcer.

**Figure 2:**
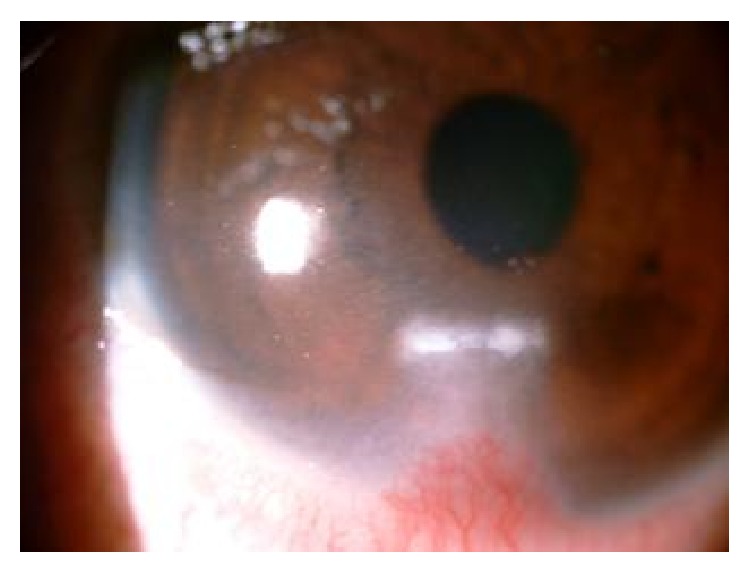
Healed corneal ulcer after treatment.

A 40-year-old man, clerk by occupation, came with symptoms of pain, photophobia, watering, and blurriness of vision in his right eye of 2-month duration. He was in good general health and had no history of any systemic diseases, recent ocular trauma, ocular surgery, or contact lens use. He was diagnosed elsewhere with nonhealing corneal ulcer and was on topical fluoroquinolone ofloxacin and antifungal fluconazole eye drops. However his condition showed no improvement. His earlier reports showed positive Mantoux test with induration of 18 mm done 1 month back and normal blood sugar levels and seronegativity for human immunodeficiency virus (HIV) and Hepatitis B and Hepatitis C virus. TB gold test was negative. Upon presentation, best corrected visual acuity (BCVA) was 0.48 Log MAR in the right eye and 0.00 Log MAR in the left eye, respectively. Slit lamp biomicroscopy in the right eye revealed a paracentral 3 mm *∗* 3 mm horizontally oval corneal ulcer from 5 to 7 o'clock meridian inferiorly 2 mm away from limbus with underlying stromal edema (Figure 1). Corneal sensations over the ulcer were normal. Circumcorneal congestion was present. There were no satellite lesions. It was a whitish dry looking ulcer. Anterior chamber showed mild reaction with 1+ cells and no hypopyon. The ulcer stained positively with fluorescein. The right pupil was reacting sluggishly to light in view of prior use of homatropine eye drops which patient had stopped 1 week ago. Fundus showed mild vitreous haze with no focal retinal or choroidal pathology. Intraocular pressure was 18 mm of Hg on noncontact tonometry. The lacrimal sac bilaterally showed no regurgitation on pressure and syringing showed patent lacrimal passage. B scan ultrasonography was done. It was essentially normal with no evidence of scleritis. Systemic examination revealed no lymphadenopathy and clear respiratory system. Scraping was done with number 15 blade and sent for microbiological examination. All microbiological tests were done in a well-equipped laboratory with trained personnel. Gram staining revealed gram positive, irregular stain and forms of* M. chelonae*. ZN staining revealed acid fast bacilli, red magenta rods typical of* M. chelonae*. Further culture on LJ media at 1 week revealed characteristic* M. chelonae* colonies, circular, smooth, pale cream colonies. To identify the microorganism, its phenotypic characteristics were used, such as pigmentation of colonies growing in the darkness on LJ media-white, cream pigment production (nonchromogenic). Iron reuptake test was negative. Identification to species level was achieved on the basis of the growth characteristics, including growth in less than 7 days, growth at 37°C, failure of growth in presence of sodium chloride 5%, pigment production, and positive catalase test. Antibiotic sensitivity with Kirby-Bauer disc diffusion method showed positive sensitivity to amikacin. Patient was started on topical fortified amikacin (50 mg/mL) and topical tobramycin (14 mg/mL) with rapid resolution of symptoms and healing of ulcer in 3 weeks (Figure 2) leaving behind a nebulomacular corneal opacity in the involved area with BCVA improving to 0.18 at 1 month after treatment.

## 3. Discussion

Spontaneously occurring* M. chelonae* infection presenting as a nonhealing corneal ulcer in a healthy young adult with no predisposing factor has not been reported till date. Nontuberculous mycobacteria (NTM) are aerobic, non-spore-forming, nonmotile acid fast bacilli. Runyon proposed a classification of nontuberculous mycobacteria, under which group IV is the* M. fortuitum* complex, consisting of* M. fortuitum*,* M. chelonae*,* M. smegmatis*, and* M. vaccae* [7]. Nontuberculous mycobacteria have been isolated from the normal flora of human sputum, gastric contents, and ocular surfaces and are ubiquitous in soil, animals, milk, foodstuffs, tap water, and laboratory water [1]. Both* M. fortuitum* and* M. chelonae* are responsible for a growing number of skin and soft tissue diseases. NTM as a cause of nonhealing corneal ulcers is also well established. Girgis et al. in their retrospective study of ocular infections caused by nontuberculous mycobacteria reported 36.6% incidence of keratitis most commonly caused by* M. abscessus*/*chelonae* with identifiable risk factors being presence of biomaterials (63.1%), ocular surgery (24.1%), and steroid exposure (77%) [8]. Feder et al. reported a case of concurrent unilateral* M. chelonae* keratitis and canaliculitis in a patient with a SmartPLUG [3]. Chandra et al. [9] and Freitas et al. [10] reported cluster of cases developing* M. chelonae *keratitis following bilateral simultaneous LASIK with successful treatment with topical amikacin, azithromycin, and ciprofloxacin in most cases. Surgical debridement and flap removal were required in some. Yamaguchi et al. reported 39 eyes of 30 patients developing* Mycobacterium* keratitis following LASIK [11]. Lalitha et al. reported 6 of the 18 cases of nontuberculous mycobacterial ocular infections presenting as corneal ulcers with sensitivity to gentamicin and amikacin [12]. Huang et al. in their retrospective review of 22 cases of NTM keratitis revealed a causal history of trauma in 18 cases, ocular surgery in 2 cases (after pterygium excision and after failed corneal graft) [13]. They found one case with ocular surface disease in a patient of cicatricial pemphigoid and one case after neuropathic keratopathy following cerebellopontine angle tumour removal. Malecha and Doughman reported a case of* M. chelonae* keratitis associated with soft contact lens usage [14]. Siong and Felipe reported 13 cases of ocular NTM infection after phacoemulsification cataract surgery of which 77% presented with stromal wound abscesses [2]. Keratitis is the most common type of ocular NTM infection, most commonly caused by rapid growers* M. chelonae* and* M. fortuitum*. Typical “cracked windshield” appearance of cornea is considered diagnostic [13]. Most of these cases have a history of preceding ocular trauma usually with metallic foreign body or prior history of ocular surgery, contact lens wear, or systemic immunosuppression. Due to unpredictable response to topical antibiotic therapy and frequent need of surgical intervention like lamellar keratectomy and penetrating keratoplasty, it leads to significant visual morbidity [9, 10]. Early recognition and prompt institution of appropriate antibiotic in accordance with antibiotic sensitivity testing are important. Identification and culture of the organism from corneal scrapings form the basis of definitive diagnosis. Of the mycobacterial family,* M. chelonae* is mostly sensitive to tobramycin, whereas* M. abscessus* is not and is more sensitive to fluoroquinolones. We report on a case of spontaneously developed ZN smear and culture positive active NTM ocular infection with focus on cornea presenting as a nonhealing corneal ulcer with no known predisposing factor. Early recognition and prompt institution of topical amikacin and tobramycin prevented the ocular morbidity. Till date,* M. chelonae* keratitis has been reported consequently to some trauma or iatrogenic intervention or in immunosuppressed states. However, the possibility of incidental environmental inoculation cannot be ruled out. The present case stresses the importance of considering* M. chelonae* in the differential diagnosis of nonhealing corneal ulcer even in nonpredisposed healthy adults with no obvious history of ocular trauma and the role of subsequent early institution of appropriate sensitive drugs in preventing the consequent visual morbidity and the need of surgical intervention.
